# Assessing Quality in Interventional Practice: Identifying Meaningful and Actionable Metrics

**DOI:** 10.31083/j.rcm2502058

**Published:** 2024-02-05

**Authors:** Lloyd W Klein

**Affiliations:** ^1^Cardiology Section, University of California, San Francisco, San Francisco, CA 94117, USA

**Keywords:** PCI, TAVR, quality assessment

## Abstract

Interventional cardiologists should insist on quality assessment techniques that indisputably reflect the merit of care delivered. Only measurable outcomes and metrics that are modifiable should be identified and collected. An evaluation process should be adopted that genuinely appraises clinical practice, incorporating appropriate benchmarks for comparison.

## 1. Introduction

The accurate evaluation of the quality of the performance of interventional procedures is vital to delivering optimal patient care and clinical outcomes [1, 2, 3]. Procedural volume and in-hospital mortality are certainly important as initial indicators of the efficacy of a procedure and also are simple to obtain. However, these data are relevant only to the short term; there is no evaluation of long-term outcome, information is not provided about results in specific patient subsets and thus cannot reflect improvable deficiencies that can be addressed and perfected. Instead, they have become weaponized to critique individual operators or programs, because the precise systemic conditions leading to poor results are not collected [2, 3]. Consequently, clinicians have been incentivized to find workarounds to current quality indicator collection, as fault is ascribed to the operator, which is usually undeserved.

## 2. Common Coronary Metrics

The standard clinical quality metric in coronary intervention is unadjusted 30-day survival [3, 4]. If a death occurs within that time frame, the interventional procedure is considered its cause, regardless of whether or not a distinguishable complication occurred. No modification of fault assignment is available despite the reality that neither the operator nor the program can alter the risk characteristics of the patient they are treating. Thus, variables that are determinants of short-term mortality, such as acute infarct size, cardiogenic shock, time to emergency department arrival (ED) and cath lab arrival after acute symptom onset, the severity and extent of coronary artery disease or the presence of comorbidities, are not taken into account when assigning culpability. Consequently, 30-day mortality is an inadequate measure of the skills of the interventionist or the strength of the interventional program.

This measure is very popular for public reporting because of its apparent simplicity, but it is not a strong metric of quality. The reason is that most deaths within 30 days of an interventional procedure are more closely associated with the clinical situation than the intervention itself. Moreover, public reporting of raw mortality data encourages risk aversion. The consequent intentional selection of very low-risk patients for procedures diminishes its value and makes performance measures subject to gaming, leading to further inaccuracy [5]. And, performing high-risk cases in high volume may incorrectly result in criticism of those taking on cases with the worst intrinsic prognosis. Since the highest clinical benefit is derived from intervention in high-risk conditions, the consequences run counter to our purpose as physicians. The expected event rate is higher, and deaths are inevitable, independent of the competence of the team, leading to unwarranted damage to professional reputation [1, 2, 3].

Risk adjustment algorithms to adjust for these confounders have been developed. The 30-day risk-adjusted mortality rate (RAMR) is calculated as the observed to expected mortality ratio (the number of observed to expected (O:E) deaths) multiplied by the patient-level average mortality rate (about 1.3%). Expected mortality is calculated by an adjustment algorithm from a weighted formula comprised of comorbidities associated with worse outcomes [4, 6]. However, risk adjustment algorithms only partially correct for risk because any mortality leaves a minor O:E fraction, since observed mortality (the numerator) can never be zero. This residual accumulates cumulatively with each additional mortality; so the inherent inaccuracy increases with each subsequent event. Hence, the more high-risk cases that are performed, the more inaccurate the O:E ratio [7], regardless of expected mortality. For this reason, risk adjustment is imprecise at the high end of risk [8].

Another limitation is that risk adjustment models do not include all of the factors used to make clinical decisions. These unmeasured confounders often have powerful associations with expected outcomes. Many experts advocate excluding cases with a high intrinsic risk from analysis. For example, they would exclude cases with cardiogenic shock entirely, and have a separate algorithm and analysis for acute ST elevation myocardial infarction [2]. This suggestion has not been initiated despite a decade and a half of calls for reform. The primary reason is that when high-risk cases are excluded from the analysis, the resulting observed mortality rate is very low [6]. This would render distinguishing low-quality results mathematically impossible, rendering the exercise valueless, and making the data collection unhelpful despite the expense. Post-procedural death in usual-risk patients is usually related to events of bleeding, heart failure, arrhythmias, renal failure, and patient frailty, rather than the technical and cognitive skill of the operator. So that there is something to measure, these occurrences are themselves counted as quality measures. This practice further distorts quality measurement, as it is usually outside the interventionists’ control to select cases with a low likelihood of complications.

Case volume continues to be used as a measure of quality despite years of recognition that it is a highly simplistic and inaccurate measure for this purpose [2]. Surely a learning curve for any procedure exists; but once that volume is attained and maintained, further reliance on quantity should no longer correctly predict outcomes or complications. The use of percutaneous cardiology intervention (PCI) operator volume as a measure of quality is thus inexact, and analysis shows it is only very weakly correlated, when it correlates at all, with outcomes [1, 2, 3]. Rather than take the proper but politically unpalatable approach of training the correct number of specialists and superspecialists, arbitrary volume minimums are created without supportive data [8]. Although a common belief persists that the number of cases an operator performs correlates with patient outcomes, most of the objective evidence fails to support this contention [1, 2, 3]. This has resulted in imposing arbitrary volume requirements for certification, which are frequently higher than many practitioners can achieve [8]; yet there is no practical consequence for failing to meet this standard.

## 3. Additional Interventional Procedures

Transcatheter Aortic Valve Replacement (TAVR) and Transcatheter Edge-to-Edge Repair (TEER) have become routine interventional procedures. Important quality metrics for these procedures should focus on evaluating their safety, efficacy, and patient outcomes. Yet 30-day mortality and case volume are most frequently utilized as key quality metrics, both in clinical practice and in multi-center trials. Although the rate of death following the procedure is a critical metric to assess safety and effectiveness, as is procedural success and complication rates (stroke, bleeding, vascular complications, valve-related issues, and heart rhythm disturbances), they are not accurate quality indicators because the same lack of risk adjustment and dependence on case selection are applicable in these procedures.

## 4. Proposed Solution

A comprehensive framework that incorporates broad aspects of practice has been proposed [9, 10]. Appraisal is conducted by measuring four parameters: case selection, technical expertise, case complexity, and clinical results. Primary quality indicators to be assessed include quality of life, occurrence of angina, re-hospitalization, repeat revascularization, and follow-up myocardial infarction. These endpoints should be the central components in revising the quality framework. Additionally, reduction of specific non-fatal but procedural-related complications, such as hematomas, bleeding, dialysis, stroke, periprocedural myocardial infarction (MI), and stent thrombosis, should be mandatory facets of the evaluation process. Appropriate selection of patients should optimally be based on the correlation of coronary stenoses with regional ischemia, function and viability should be included in the assessment. The use of physiologic testing and intracoronary imaging are necessary to fully optimize strategy and results. Physiologic testing is underutilized to identify significant lesions [11]. Intravascular imaging to guide the performance of the procedure has been shown to lead to improved outcomes in the RENOVATE-COMPLEX-PCI trial [12]. Treatment selection that optimally incorporates patient preference must be integrated into the appraisal process. Other important elements include independent case review and comparison of outcomes to a disease-specific registry.

For TAVR and other valvular corrections, collected data should include:

∙ Length of Hospital Stay: after TAVR or TEER.

∙ Quality of Life Improvement: Evaluating how the procedures impact the patients’ quality of life and functional status.

∙ Hemodynamic Performance: Assessing the improvement in heart valve function and hemodynamics after the procedure is critical to determine efficacy.

∙ Valve Durability: Tracking the long-term durability and function of the implanted valve.

∙ Repeat Procedures: The need for repeat procedures, including valve-in-valve, is an important quality indicator.

∙ Follow-up Rates: Ensuring patients receive appropriate post-procedural follow-up care.

∙ Patient Satisfaction: Measuring patient satisfaction and understanding their experiences.

∙ Stroke that is unrelated to the procedure should not be used as an indicator.

Evaluating these metrics routinely would ensure that TAVR and TEER procedures are performed safely and effectively, and provide patients with improved outcomes and quality of life.

## 5. What Needs to be Done Now

A large part of the reason quality programs have not incorporated these important but “soft” measures is that they are costly and time consuming to collect, and there is no motivation for individual programs to allocate its scarce resources to such a project. Why should resources be expended in knowing what our results are and what benefit is it to us or our patients to spend our time collecting these results? The whole point of quality assurance programs is to correct things that can be improved to make our patients live longer and better. If our profession expects to demonstrate its value and expected outcomes, as it must to justify societal expenditures and need for healthcare, then it is our responsibility to define the parameters and collect them.

The only way to develop such an approach would be universal mandatory data collection. If structured reporting with universal data collection became mandatory, then transitioning to a modern and useful quality assurance program would be facilitated. Instead of directed to place “fault” on physicians and programs, the purpose would be to improve the patient experience and outcomes by correcting deficiencies identified objectively. Aggregated data is a potentially powerful tool for benchmarking; allowing comparison with other similar programs in one’s geographic area not the largest national centers. Structured reports, centered on a single-database cardiology solution, offer numerous benefits, including improved clinical efficiency, diagnostic accuracy, and resource management. By automating data entry and streamlining workflows, structured reporting would simplify the process of submitting data to registries and accreditation bodies.

Successful adoption would require buy-in from executive leadership, IT, and clinical users; and that is why insisting on mandatory universal adoption is necessary. If the structured reporting solution were designed to be clinically robust, user-friendly, and improve clinical workflow, adoption would meet little resistance from any stakeholder. If implemented correctly, structured reporting raises the possibility of improving clinician data consumption and accuracy, making quality assessment not a tool designed to critique operators but rather to elevate standard of care and service delivery.
